# The effects of mineral trioxide aggregate and second-generation autologous growth factor on pulpotomy via TNF-α and NF-kβ/p65 pathways

**DOI:** 10.1186/s12903-024-04577-z

**Published:** 2024-08-03

**Authors:** Ayça Kurt, Ahter Şanal Çıkman, Emre Balaban, Zeynep Gümrükçü, Tolga Mercantepe, Levent Tümkaya, Mert Karabağ

**Affiliations:** 1 Department of Pediatric Dentistry, Faculty of Dentistry, Recep Tayyip Erdogan University, Rize, 53100 Turkey; 2 Department of Endodontics, Faculty of Dentistry, Recep Tayyip Erdogan University, Rize, 53100 Turkey; 3 Department of Oral and Maxillofacial Surgery, Faculty of Dentistry, Recep Tayyip Erdogan University, Rize, 53100 Turkey; 4 Departments of Histology and Embryology, Recep Tayyip Erdogan University, Rize, 53100 Turkey

**Keywords:** Concentrated growth factor, Inflammation, Mineral trioxide aggregate, NF-kβ/p65, Pulpotomy, TNF-α

## Abstract

This study aims to investigate the effect of Mineral Trioxide Aggregate (MTA), a bioactive endodontic cement, and Concentrated Growth Factor (CGF), a second-generation autologous growth factor, on pulpotomy-induced pulp inflammation. The study utilized the maxillary anterior central teeth of thirty-six young male Sprague Dawley rats. Forty-eight teeth were randomly assigned to two groups (12 rats/group; 24 teeth/group) based on the capping material (MTA or CGF). Subsequently, two subgroups (MTAG and CGFG) were formed per group (12 teeth/group) based on the time following pulpotomy (2-weeks and 4-weeks). The central teeth of the 12 animals assigned to the control group (CG) were not manipulated in any way, both in the 2-week group and in the 4-week group. Tissue samples extracted from rats at the end of the experiment were stained with H&E for histopathological analysis. For immunohistochemical analysis, primary antibodies for TNF-α and NF-kβ/65 were incubated. Data obtained from semi-quantitative analysis were assessed for normal distribution using Skewness-Kurtosis values, Q-Q plot, Levene’s test, and the Shapiro-Wilk test on statistical software. A P value < 0.05 was considered significant. When compared with the control group, both MTAG and CGFG showed increased edematous and inflammatory areas. In MTAG, edematous and inflammatory areas decreased significantly from the 2nd week (2(2–2), 2(1–2)) to the 4th week (1(1–1), 1(0–1)), while in CGFG, edematous areas decreased (2(2–3), 1.5(1–2)), and inflammatory areas increased significantly (2(2–3), 3(2-2.5)). When compared with the control group, TNF-α and NF-kβ/p65 positivity were higher in both MTAG and CGFG. In MTAG, TNF-α [2(1.5-2)] and NF-kβ/p65 [1.5(1–2)] positivity decreased significantly from the 2nd week to the 4th week [TNF-α: 1(1–1), NF-kβ/p65: 1(1–2)], while no significant change was observed in CGFG. In conclusion, this study revealed a reduction in cells showing TNF-α and NF-kβ/p65 positivity in the MTA treatment group compared to the CGF group. Although MTA demonstrated more favorable results than CGF in mitigating pulpal inflammation within the scope of this study, further experimental and clinical investigations are warranted to obtain comprehensive data regarding CGF.

## Introduction

Caries are recognized as a preventable chronic health issue. However, comprehensive global reports [1] suggest that in 2015, there were approximately 573 million children with untreated caries [2]. Although caries predominantly affect young children, it persists as a lifelong condition encountered during adolescence and adulthood. Therefore, the treatment of caries, particularly deciduous caries, is of paramount importance [3].

The preservation of pulp is crucial during the treatment of complex root canal systems in deciduous molar teeth [4, 5]. Exposure to pulp tissue can occur due to caries, iatrogenic causes, or traumatic injuries. Pulpotomy, a widely used procedure in pedodontics, is effective in preserving deciduous teeth [6, 7]. It involves the removal of a portion of the coronal pulp tissue and capping of the radicular tissue with materials that prevent further injury to the pulp and promote healing [8].

Various materials have been employed to stimulate the formation of a dentin bridge through the dentinogenic potential of pulp cells [9]. The growing emphasis on biocompatible materials has led to a wider range of Mineral Trioxide Aggregate (MTA) options for pulpotomy. MTA, which protects periradicular or dental pulp tissues, can preserve pulp vitality and aid in its recovery [10]. Despite its high success rates, MTA has drawbacks such as pulp chamber obliteration, tooth discoloration, pulp degeneration, and cost concerns [11]. Hence, there is a need for the development of biocompatible treatments aimed at protecting pulp vitality and enhancing tooth survival [7].

Concentrated Growth Factor (CGF), a second-generation autologous growth factor, is a recently discovered platelet concentrate derived from autologous venous blood. It produces a larger and denser fibrin matrix compared to previously known platelet concentrates [12]. Acting as a scaffold material, CGF also releases numerous growth factors at the site of application [13]. Despite extensive research on CGF, its direct application as a pulpotomy material has not been investigated [12, 14–18]. Bio-compatible content and the beneficial combination of numerous growth factors and chemotactic factors make CGF a promising biomaterial in clinical pulp injury applications, as it may promote pulp regeneration through its excellent regulatory properties in inflammation, proliferation, migration, and odonto/osteogenic differentiation [18, 19]. As a platelet concentrate, CGF holds promise as a scaffold for the treatment of dentin-pulp complex disorders. It is known that CGF, as a natural biomaterial, contains platelets, cytokines, and growth factors to facilitate the healing process [20, 21], yet based on the information obtained from the literature review, we can say that its use as a pulpotomy material is being explored for the first time in this current pilot study.

Although MTA and CGF are both considered regenerative therapeutic agents in pulpotomy treatment, the MTA material lacks the fibrin matrix containing growth factors that CGF possesses [22]. CGF, with this feature, acts as a scaffold in pulpal treatments, enhancing cellular proliferation, differentiation, and angiogenesis, serving as a matrix for tissue growth, and regulating inflammatory reactions [18]. All these properties make CGF advantageous compared to MTA. Additionally, its highly biocompatible nature and derivation from the patient’s blood offer advantages over the expensive MTA [23].

Similar to other types of connective tissue, the inflammatory response in dental pulp is possible through the activation of the immune system, including macrophages. Once activated, macrophages secrete pro-inflammatory cytokines such as tumor necrosis factor-alpha (TNF-α). Cytokines; are regulators of host responses to infection, immune responses, inflammation, and trauma [24]. TNF-α; are pro-inflammatory cytokines, and their increase in the environment stimulates osteoclastic activity, which impairs the inhibition of osteoblast activity and apoptosis of osteocytes [25]. Nuclear factor kappa beta (NF-kβ) regulates the production of some cytokines that stimulate inflammatory reactions. NF-kβ is one of the main regulators of inflammatory cytokines, and the most common transcription factors are p50 and p65 [26]. Nuclear factor kappaB/p65 (NF-kβ/p65) is a versatile transcription factor that can affect the transcription of many genes. It plays an important role in physiological and pathological processes such as immune response, inflammatory response, cell differentiation and apoptosis [27]. In the pro-inflammatory cascade, TNF-α activates NF-kβ/p65 transcription pathways, which in turn lead to exacerbation of problems including infection and autoimmune diseases. Research on tumor necrosis factor-alpha (TNF-α) and NF-kβ/p65 is tightly intertwined. Cytokines belonging to the TNF-α family induce rapid transcription of genes that regulate inflammation, cell survival, proliferation, and differentiation, primarily through activation of the NF-kβ pathway [28]. A few studies examine the relationship between TNF-α and NF-kβ/p65 in the inflammatory pathogenesis that may occur in the pulp, induced by pulpotomy [23, 29].

This study aims to explore alternative materials for pulpotomy treatment in endodontic procedures by investigating the potential effects of inflammation resulting from the use of MTA, a bioactive endodontic cement, and CGF, a second-generation autologous growth factor, as pulpotomy materials, on the TNF-α and NF-kβ/p65 pathways.

## Materials and methods

### Ethics approval

The required ethics committee decision for the study was granted by Recep Tayyip Erdogan University, Faculty of Medicine, Animal Research Ethics Committee (approval number: 2020/47). All methods are reported by ARRIVE guidelines (https://arriveguidelines.org//).

### Experimental design

The study used the maxillary anterior central teeth of thirty-six young male rats (Sprague Dawley, weighing between 250 and 350 g). The maximum sample size of this study was chosen to be in line with the studies of Arifin et al. [30]. The sample size was calculated with the formula DF = N – k = kn – k = k(n – 1) (total number of subjects, k = number of groups, and n = number of subjects per group [30]. In total, 72 maxillary central teeth were included in the study. We randomly assigned 48 teeth to two groups (12 rats/group; 24 teeth/group) based on the capping material (MTA or CGF). Two subgroups per group were formed (12 teeth/group) based on the time following pulpotomy (two weeks and four weeks). The central teeth of the 12 animals assigned to the control group were not manipulated in any way, both in the 2-week group and in the 4-week group; thus, these teeth served as the control group for histological identification. Throughout the experiment, the animals were housed in polypropylene cages (with four animals per cage) in an experimental animal unit with a 12:12-hour light-dark cycle, 55–60% humidity, and room temperature, as well as unlimited availability of water and food pellets (Nutrilabor; Guabi, Campinas, SP, Brazil). The cage bedding was renewed three times per week at a minimum.

### Operational procedure

The animals received anesthesia via intramuscular injection of a mixture containing 50 mg/kg (i.p) ketamine hydrochloride (Ketalar ^®^, Eczacıbaşı Parke-Davis, Istanbul, Turkey) and 10 mg/kg (i.p) Xylazine HCl (Alfazyne ^®^, Alfasan International B.V. Woerden, The Netherlands) and were immobilized on a surgical board.

For pulpotomy, the conventional technique was followed. The permanent maxillary right and left central incisors were isolated as previously described [22, 31]. A round diamond bur (#1011, Dentsply Indústria e Comércio Ltda, Petropólis, RJ, Brazil) and a high-speed rotary handpiece were used to create an opening for pulpotomy, forming a 1 mm cavity in the middle third of the crown in the palatal part of the incisors. The coronal pulp tissue was removed using an excavator (#5, SS White, Dental Objects Ltda., Juiz de Fora, MG, Brazil), and bleeding in the pulp chamber was controlled with saline-soaked cotton pellets. Loupes with illumination (Loupes, YDL-EW, 2.5x) were used to enhance the field of view. Before the pulpotomy procedure, the mucosa of the central teeth was anesthetized with infiltrative local anesthesia using articaine (ULTRAVER D-S), containing 80 mg articaine hydrochloride and 0.01819 mg epinephrine bitartrate.

Animals in the control group (CG) were also divided into two subgroups (2-weeks and 4-weeks). These animals underwent immobilization with appropriate anesthesia and local anesthesia, despite no dental procedures being performed. Tissue samples were collected in the 2nd and 4th weeks, following previous studies focusing on odontoblastic regeneration in tissue damage caused by pulpotomy [22, 32–34].

In the experimental groups, either MTA or CGF was used as the pulpotomy material. In the MTA group (MTAG), a 1:1 mixture of MTA powder and distilled water was placed in the pulp chamber using an MTA carrier (MAP One, Produits Dentaires SA, Switzerland). In the CGF group (CGFG), 4 ml of blood was collected from the jugular vein of each rat and placed in CGF kit tubes with 0.025 M calcium chloride (S6M, STR Biotechnologies, Çorum, Turkey). The tubes were centrifuged according to a standard procedure, resulting in the formation of four layers. The gel-like CGF layer was extracted, cut from the red blood cell layer, and placed onto the exposed pulp tissue using a sterile handpiece (Medisana double-ended dental spatula). Cavities in both groups were restored and capped with conventional glass ionomer cement (Ketac-molar, ESPE Platz, Seefeld, Germany). The animals were observed during healing, with potential changes in feeding, drinking cycles, and behavioral profiles evaluated.

After the experimental period (2nd and 4th weeks after pulpotomy), the rats were sacrificed by administering sevoflurane (Sevorane 100%, Abbott, United States of America).

### Histopathological analysis procedure

Maxillary tissue specimens obtained from the rats were fixed by immersion in 10% phosphate-buffered neutral formalin (Sigma-Aldrich, Germany, Ph:7.4) for 24 h. After fixation, these specimens were decalcified by immersion in Morse’s solution for 24 h. Subsequently, the specimens underwent dehydration using an ethanol series with gradually increasing concentrations (50%, 70%, 80%, 90%, 96%, and 100%, Merck GmbH, Germany) in an autotechnicon device (Citadel 2000, ThermoScientific, Germany), followed by clearing and paraffin inclusion procedures using paraffin (Merck GmbH, Germany).

Maxillary tissue specimens embedded in hard paraffin (Merck GmbH, Germany) were sectioned into 4–5 μm slices using a rotary microtome (RM2525, Leica Biosystems, Germany). The sections were then stored in an incubator and subjected to two series of xylene (Merck GmbH, Germany) to remove paraffin. After deparaffinization, the sections were stained with Harris hematoxylin (Merck, Germany) and Eosin G (Merck, Germany) stains using a staining device. Stained sections were mounted with Entellan (Merck GmbH, Germany) and photographed under a light microscope equipped with a digital camera (BX51, Olympus Corp., Japan).

### Immunohistochemistry (IHC) analysis procedure

To examine the maxillary inflammation induced by the invasive procedure, primary antibodies against TNF-α (ab220210, Abcam, UK) and NF-kβ/p65 (ab16502, Abcam, UK) were utilized, along with corresponding secondary antibody kits (Goat Anti-Rabbit IgG H&L (HRP), ab205718, Abcam, UK).

Sections of 1–2 μm thickness were obtained from maxillary tissue sections using a rotary microtome (Leica Biosystem, RM2525, Germany). The obtained sections were then incubated with primary and secondary antibodies for one hour, following the manufacturer’s guidelines provided in the catalogue.

Immunostaining was performed using the immunoperoxidase method with an IHC staining device (Bond Max III, Leica Systems, Germany). Finally, the sections were counterstained with Harris hematoxylin (Merck, Germany) for visualization.

### Semi-quantitative analysis

Harris hematoxylin (Merck GmbH, Germany) and Eosin G (Merck GmbH, Germany) -stained maxillary tissue sections were given a histopathological damage score (HDS) by the studies that evaluated the indications caused by invasive procedures of the maxilla concerning edema, polymorphonuclear leukocyte inflammation, fibrosis, as shown in Table 1. The tissues in both the coronal and radicular parts of the dental pulp were examined. From each preparation, 20 different randomly selected sites were scored by a histopathologist under a light microscope using x21 and x20 objectives.

**Table 1:** Histopathological damage score (HDS)

Findings	Score
Edema	
0	Less than 5%
1	Between 6-25%
2	Between 26-50%
3	More than 50%
Inflammation	
0	Less than 5%
1	Between 6-25%
2	Between 26-50%
3	More than 50%

Immunohistochemical analyzes (IHC); specimens of maxillary tissue incubated with TNF-α and NF-kβ/p65 primary antibodies were scored as shown in Table 2. From each preparation, 20 different randomly selected sites were scored by a histopathologist under a light microscope using x40 objectives. The histopathologists were blind to the experimental groups.

**Table 2:** Semi-quantative analysis Scoring methods

Findings	Score
0	Less than 5%
1	Between 6-25%
2	Between 26-50%
3	More than 50%

### Statistical analysis

Data obtained from the semi-quantitative analysis were evaluated for normality of distribution using the Skewness-Kurtosis values, Q-Q plot, Levene’s test, and the Shapiro-Wilk test on the SPSS 20 (IBM, USA) statistics software. For non-parametric data, median-25% and 75% interquartile range were calculated and analyzed with the Kruskal Wallis test followed by Tamhane’s T2 test. P value < 0.05 was accepted as significant.

## Results

### Histopathological findings

**Fig. 1:**
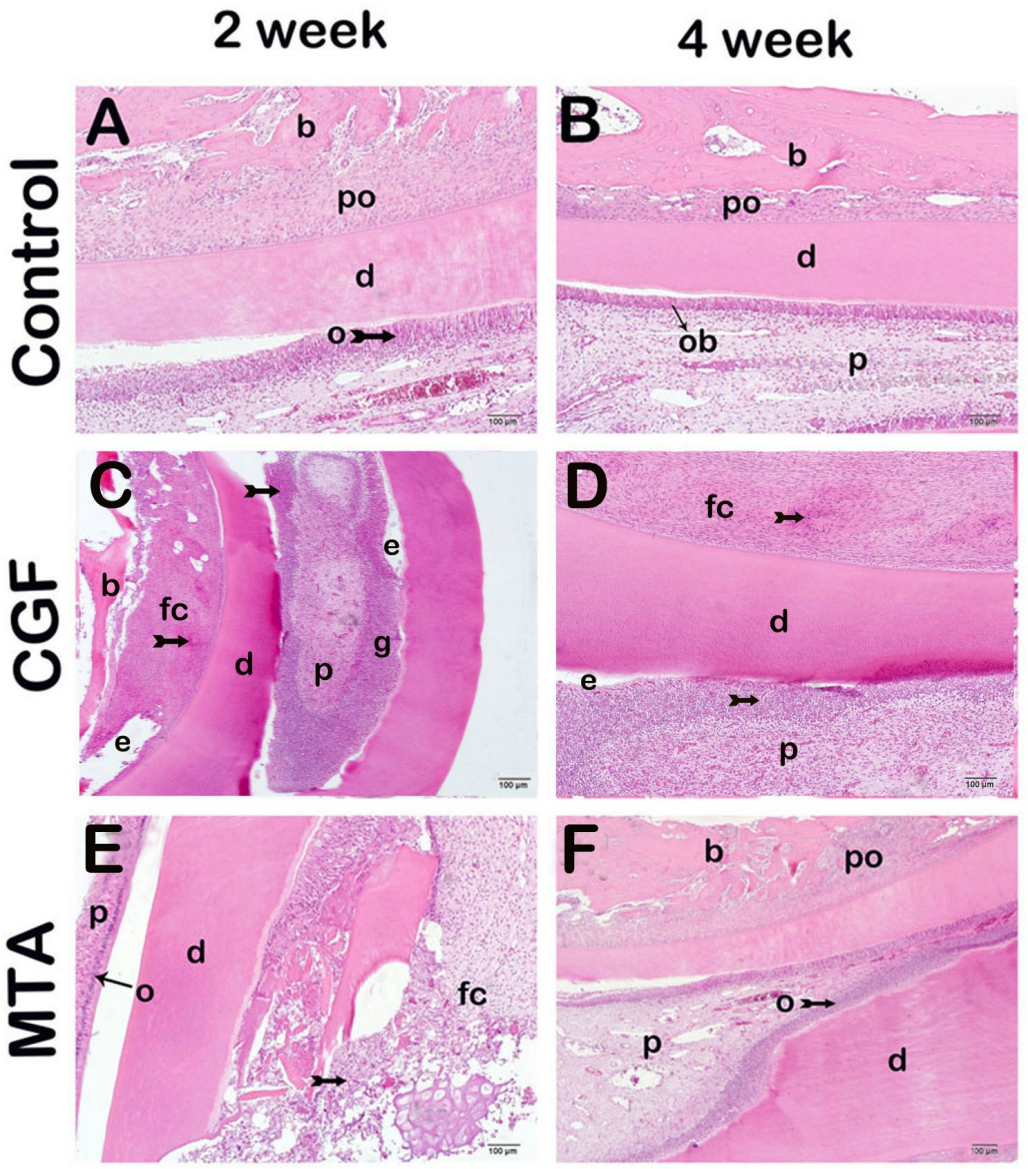
Representative light microscopic screenshot of maxillary sections stained with H + E. bone (b), dentin (d), edematous areas (e), fibrous callus (fc), periodontal ligament(po), odontoblast(o or ob), pulp(p) **A(x20) CG 2 W**: Dentin (d), periodontal ligament (po), alveolar bone (b) and pulp structures formed by normal odontoblasts (o or ob) are observed (HDS: 0.5(0–1)). **B(x20) CG 4 W**: The maxilla, which consists of dentin (d), periodontal ligament (po), alveolar bone (b) and pulp (p) structures formed by normal odontoblasts (ob), is observed (HDS:1(0–1)). **C(x20) CGFG 2 W**: Widespread inflammation (tailed arrow) accompanied by granuloma tissue (g) and widespread edematous areas (e) are observed in the application areas (HDS: 5(4–5)). **D(x20) CGFG 4 W**: Inflammation (tailed arrow), edematous areas (e) and fibrous callus (fc) are observed in the application areas (HDS:4(3–4)). **E(x20)MTAG 2 W**: A decrease is observed in infiltrative and edematous areas. In addition, diffuse fibrous callus (fc) are observed (HHS:2.5(3–4)). **F(x20)MTAG 4 W**: A decrease is observed in infiltrative and edematous areas. In addition, odontoblasts (ob), the pulp (p), periodontal ligament (po) with typical structures are observed in places. HDS: 2(2(1–2))

Histopathological findings in the crown pulp and cervical pulpal area damaged because of pulpotomy were evaluated. In the evaluation of maxillary tissue sections stained with Harris hematoxylin and Eosin G, normal structures including odontoblasts, dentin, pulp, alveolar bone, and periodontal ligaments were observed in sections from the 2-week CG and four-week CG groups (Fig. 1a-b, HDS: 0.5(0–1) and 1(0–1), respectively). However, sections from the 2-week CGF treatment group exhibited extensive inflammation accompanied by edematous areas (Fig. 1c, HDS: 5(4–5)). In sections from the 4-week CGFG group, a decrease was noted in the extensive edematous areas and infiltrative areas, along with fibrous callus formation (Fig. 1d, HDS: 4(3–4)). The 2-week MTAG group showed a decrease in infiltrative and edematous areas, along with the formation of cartilage callus in some areas (Fig. 1e, HDS: 2.5(3–4)). Similarly, in the 4-week MTAG group, a decrease in infiltrative and edematous areas was observed, along with the presence of typical odontoblasts, dentin, periodontal ligaments, and alveolar bone structures (Fig. 1e, HDS: 2(1–2)).

The 2-week and 4-week subgroups of the control group did not show significant differences in HDS scores (Fig. 1a-b, HDS 0.5(0–1) and 1(0–1), respectively). However, a higher HDS score was observed in the 2-week CGFG group (HDS: 5(4–5)) compared to both the 2-week (HDS 0.5(0–1)) and 4-week (1(0–1)) control groups (Table 3). Similarly, a higher HDS score was noted in the 4-week CGFG group (HDS: 4(3–4)) compared to both the 2-week (HDS 0.5(0–1)) and 4-week (1(0–1)) CG groups (Table 3). Conversely, lower HDS scores were observed in both the 2-week (HDS: 2.5(3–4)) and 4-week (HDS: 2(1–2)) MTAG groups compared to both the 2-week (HDS: 5(4–5)) and 4-week (HDS: 4(3–4)) CGFG groups (Table 3).

**Table 3:** Histopathological damage score results HDS, (median-25-75% interquartile range)

Group	Oedematous Area	PMNL Inflammation	HDS
CG 2 W	0(0–1)	0(0-0.5)	0.5(0–1)
CG 4 W	0.5(0–1)	0(0-0.5)	1(0–1)
CGFG 2 W	2(2–3)^a, c^	2(2–3)^a, b^	5(4–5)^a, b^
CGFG 4 W	1.5(1–2)^a,d,e^	3(2-2.5)^a, b,i^	4(3–4)^a, b,i^
MTAG 2 W	2(2–2)^a, c,f^	2(1–2)^a, b,j^	2.5(3–4)^a, b,g^
MTAG 4 W	1(1–1)^b, c,g, h,i^	1(0–1)^a, b,g, k^	2(1–2)^a, m,g, k,i^

^*a*^*p* = 0.001; versus to CG 2 W,

^*b*^*p*=0.038; versus to CG 4 W,

^c^*p* = 0.001; versus to CG 4 W,

^*d*^*p*=0.001; versus to CG 4 W,

^*e*^*p*=0.044; versus to CGFG 2 W,

^*f*^*p*=0.011; versus to CGFG 2 W,

^*g*^*p* = 0.001; versus to CGFG 2 W,

^*h*^*p*=0.046; versus to CGFG 4 W,

^*i*^*p* = 0.001; versus to MTAG 2 W,

^*j*^*p*=0.001; versus to CGFG 2 W,

^k^*p* = 0.001; ; versus to CGFG 4 W,

^j^*p*=0.002; versus to CGFG 2 W,

^*m*^*p*=0.012;versus to CGFG 2 W,

Kruskal Wallis/Tamhane T2 Test

### Immunohistochemical analysis

**Fig. 2:**
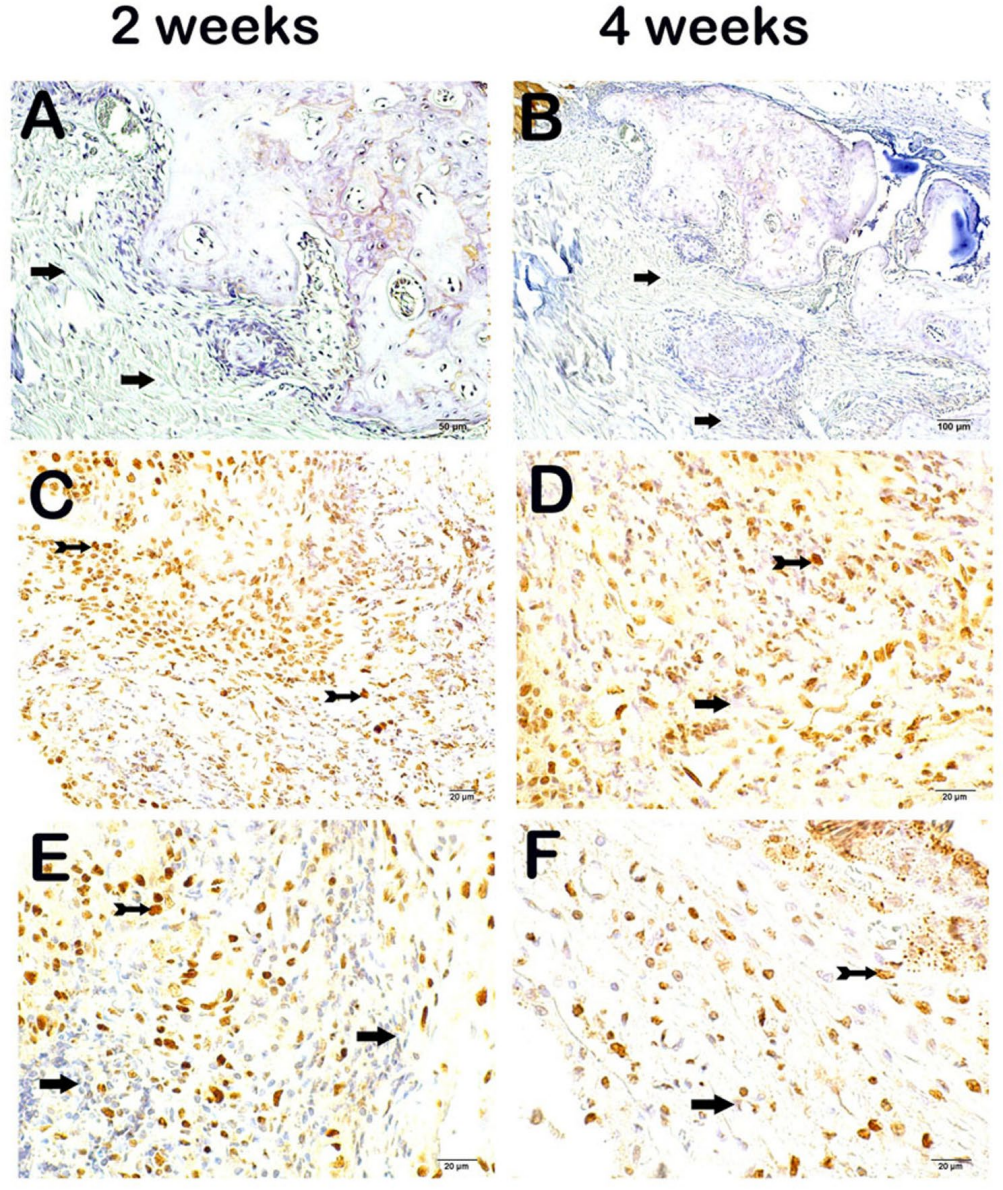
Representative light microscopic screenshot of maxillary tissue incubated with TNF-α primary antibody. **A(x40) CG 2 W**: Immune-negative cells are commonly observed in sections of maxillary tissue incubated with TNF-α primary antibody (TNF-α positivity score: 0(0-0.5)). **B(x40) CG 4 W**: Immune negative cells are commonly observed in sections of maxillary tissue (TNF-α positivity score: 0(0–1)). **C(x40) CGFG 2 W**: A large number of cells showing TNF-α positivity are observed in the sections of the maxillary tissue (TNF-α positivity score: 3(2–3)). **D(x40) CGFG 4 W**: Cells showing TNF-α positivity are observed in sections of maxillary tissue (TNF-α positivity score: 2(2–2)). **E(x40) MTAG 2 W**: In sections of maxillary tissue incubated with TNF-α primary antibody, a decrease in cells showing TNF-α positivity is observed (TNF-α positivity score: 2(1.5-2)). **F(x40) MTAG 4 W**: In sections of maxillary tissue incubated with TNF-α primary antibody, a decrease in cells showing immune positivity is observed (TNF-α positivity score: 1(1–1))

On evaluation of the maxillary tissue sections incubated with the TNF-α primary antibody under a light microscope, we observed that immune-negative cells were common in the maxillary tissues of the 2-week and 4-week control groups (Fig. 2a and b, respectively; TNF-α score: 0(0-0.5), 0(0–1)). In contrast, we determined an increase in the number of cells showing TNF-α positivity in sections from the 2-week and the 4-week CGFG compared to those from the 2-week and 4-week control groups (Fig. 2c-f respectively; TNF-α score : 3(2–3), 2(2–2) *p* = 0.001, *p* = 0.001). On the other hand, we determined a decrease in the number of cells showing TNF-α positivity in the 2-week MTAG compared to the 2-week CGFG (Fig. 2e-c respectively; TNF-α score: 2(1.5-2), 3(2–3), *p* = 0.001). Similarly, we observed fewer cells showing TNF-α positivity in the maxillary tissue sections from the 4-week MTAG compared to the 4-week CGFG (Fig. 2f-d, respectively; TNF-α score: 1(1–1), 2(2–2), *p* = 0.001). When comparing the 2-week and 4-week MTAG subgroups regarding the number of cells showing TNF-α positivity, a significant decrease was observed in the 2-week MTAG subgroup compared to the 4-week MTAG subgroup (Fig. 2e-f, respectively; TNF-α score: 2(1.5-2), 1(1–1), *p* = 0.015). In CGFG 4th week results; a decrease in immune-positive cells was observed in maxillary tissue sections incubated with TNF-α primary antibody compared to the 2nd week of CGFG (Fig. 2c-d; TNF-α positivity score: 3(2–3), 2(2–2)) (Table 4).

**Table 4:** Semi-quantitative analysis results (median-25-75% interquartile range)

Group	TNF-α Positivity Score	NF-kβ/p65 Positivity Score
CG 2 W	0(0-0.5)	0(0–1)
CG 4 W	0(0–1)	0(0–1)
CGFG 2 W	3(2–3)^a, b^	2(2–3)^a, b^
CGFG 4 W	2(2–2)^a, b^	2(2–2)^a, b^
MTAG 2 W	2(1.5-2)^a, b,c^	1.5(1–2)^a, b,c^
MTAG 4 W	1(1–1)^a, b,c, d,e^	1(1–2)^b, c,e, g^

^*a*^*p* = 0.001; versus to CG 2 W,

^b^*p* = 0.001; versus to CG 4 W,

^c^*p* = 0.001; versus to CGFG 2 W,

^d^*p* = 0.001; versus to CGFG 4 W,

^e^*p*=0.015; versus to MTAG 2 W,

^f^*p*=0.008; versus to CGFG 2 W,

^g^*p*=0.014; versus to MTAG 2 W,

Kruskal Wallis/Tamhane T2 Test

**Fig. 3:**
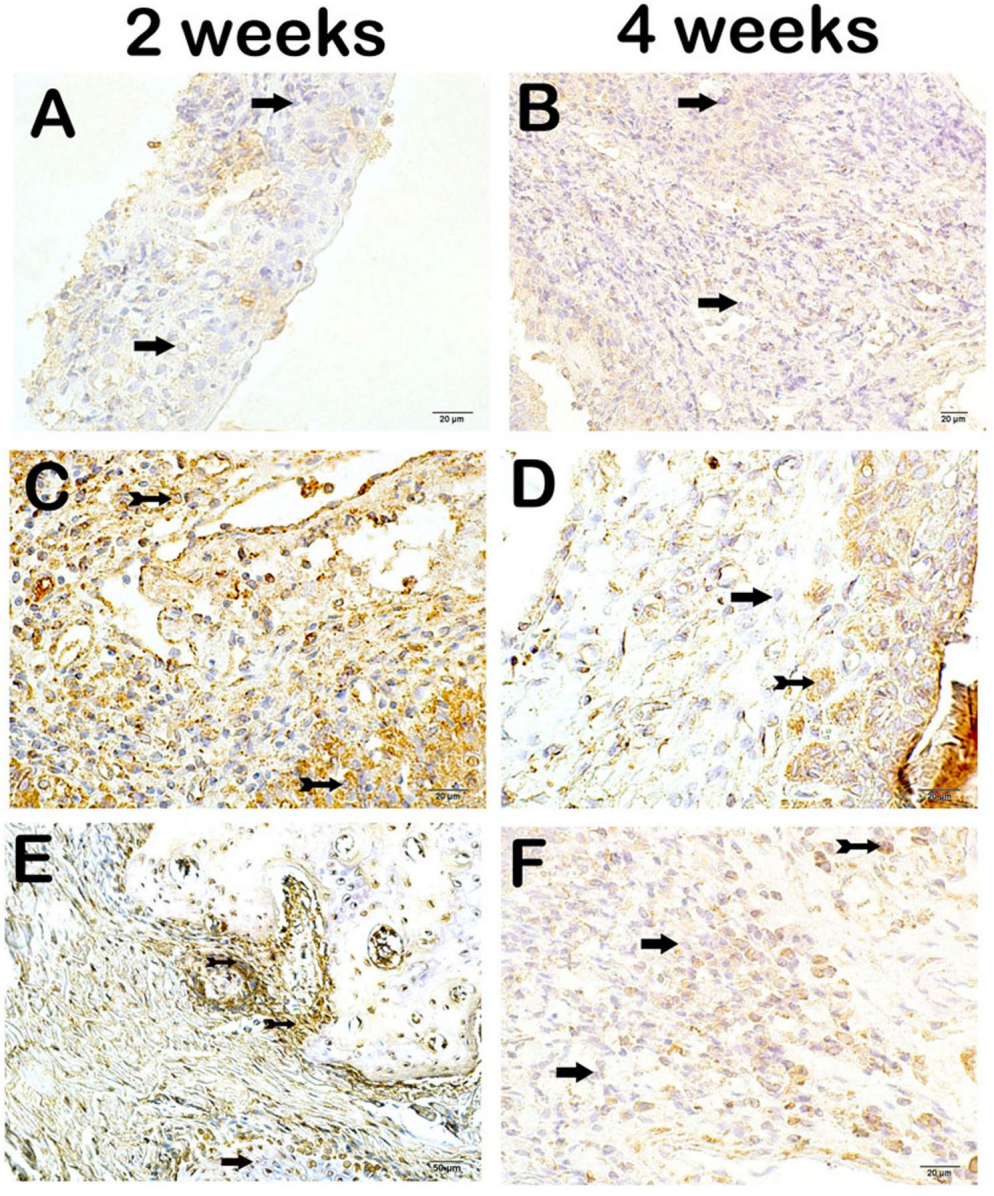
Representative light microscopic screenshot of maxillary tissue incubated with NF-kß/p65 primary antibody. **A(x40) CG 2 W**: NF-kß/p65 negative cells are commonly observed in sections of maxillary tissue (NF-kß/p65 positivity score: 0(0–1)). **B(x40) CG 4 W**: Immune-negative cells are commonly observed in sections of maxillary tissue incubated with NF-kß/p65 (NF-kß/p65 positivity score: 0(0–1)). **C(x40) CGFG 2 W**: A large number of cells showing NF-kß/p65 positivity are observed in the sections of the maxillary tissue (NF-kß/p65 positivity score: 2(2–3)). **D(x40) CGFG 4 W**: Cells showing NF-kß/p65 positivity are observed in sections of maxillary tissue (NF-kß/p65 positivity score: 2(2–2)). **E(x40) MTAG 2 W**: In sections of maxillary tissue incubated with NF-kß/p65 primary antibody, a decrease in cells showing TNF-alpha positivity is observed (NF-kß/p65 positivity score: 1.5(1–2)). **F(x40) MTAG 4 W**: In sections of maxillary tissue incubated with NF-kß/p65 primary antibody, a decrease in cells showing immune positivity is observed (NF-kß/p65 positivity score: 1(1–2))

On light microscopic examination of the maxillary tissue sections incubated with the NF-kβ/p65 primary antibody, immune-negative cells were commonly observed in the maxillary tissues of both the 2-week and 4-week CG groups (Fig. 3a-b, respectively; NF-kβ/p65 score: 0(0–1), 0(0–1)). )). Conversely, an increase in the number of cells showing NF-kβ/p65 positivity was observed in the sections from both the 2-week and 4-week MTAG groups compared to those from the 2-week and 4-week CG groups (Fig. 3e-f, respectively; NF-kβ/p65 score: 1.2(1–32, 1(1–2) *p* = 0.000, *p* = 0.001). On the other hand, we determined a decrease in the number of cells showing NF-kB/p65 positivity in the 2-week MTAG compared to the 2-week CGFG (Fig. 3e-c, respectively; NF-kβ/p65 score: 1.5(1–2), 2(2–3), *p* = 0.001). Similarly, we observed fewer cells showing NF-kβ/p65 positivity in the maxillary tissue sections of the 4-week CGFG compared with those of the 4-week MTAG (Fig. 3d-f, respectively; NF-kβ/p65 score: 2(2–2), 1(1–2), *p* = 0.015). When the 2-week and 4-week MTAG subgroups were compared to each other about the number of cells showing NF-kβ/p65 positivity, we found a significant decrease in the number of cells showing NF-kβ/p65 positivity in the 2-week MTAG subgroup compared to the 4-week MTAG subgroup (Fig. 3e-f, respectively; NF-kβ/p65 score: 1.5(1–2), 1(1–2), *p* = 0.015) (Table 4). When the 2-week and 4-week subgroups of CGFG were compared to each other concerning the number of cells showing NF-kβ/p65 positivity, there was no statistically significant change in the number of cells showing NF-kβ/p65 positivity in 2-week CGFG compared to four-week CGFG (Fig. 3c-d respectively; NF-kβ/p65 score: 2(2–3), 2(2–2), *p* > 0.05) (Table 4).

## Discussion

As there is no data in the literature regarding the effects of second-generation autologous growth factors on the tissue damage induced by pulpotomy, the present study is the first that has investigated the effects of CGF, which is a second-generation autologous growth factor, on the TNF-α and NF-kβ/p65 pathways and different intervals, in comparison to MTA, which is a bioactive endodontic cement.

Although studies investigating the tissue injuries that develop due to pulpotomy in clinics are experimental animal models, many researchers have stated that human and rat teeth have morphometric similarities [35]. Rat incisors are different from human teeth in the sense that they show continuous growth, they were proven to represent a model for the evaluation of the reactions of the human dental pulp to bioactive molecules [36, 37].

Vital pulp treatments use an appropriate pulp capping material, and this material is directly placed onto the pulp tissue; therefore, biocompatibility, anti-inflammatory properties, and cytotoxicity need to be completely evaluated before a new material is used as a pulp capping material [38]. In a study by Minic et al. [33] that investigated pulpotomy-induced pulpitis using a rat model, findings of inflammation and cellular degeneration were reported. Similarly, in the present study, we determined extensive edematous areas accompanying inflammation.

In a study conducted by Nguyen [39] the success of MTA pulpotomy in pulpitis caused by pulpotomy in primary teeth was observed. It was reported that the areas of inflammation and edema observed in the second week decreased in the fourth week. In this study, similar to previous findings, there was a decrease in edematous and inflammatory areas from the second week to the 4th week in the MTAG. Minic et al. [33]. aimed to evaluate pulp repair in rats under inflammatory conditions on the 4th and 15th days. For this purpose, they developed a controlled pulpitis rat model in which pulpotomy was performed with Biodentin, a tricalcium silicate-based cement. In the study by Minic et al. [33]. , an increase in TNF-α levels was observed after pulpotomy with Biodentin. However, no scientific studies have examined NF-kβ/p65 levels in pulpitis that develop after pulpotomy. In this current study, we observed a decrease in TNF-α levels and NF-kβ/p65 levels from the second week to the fourth week in both material groups. The differences in experimental conditions, including the duration of this study, may explain the decreasing TNF-α levels even further in the subsequent periods.

Biological scaffolds and CGF, which are known to be cytokine reservoirs, are used for bone regeneration in current clinical implantology practice [14, 40]. As a latest generation platelet concentrate product, CGF has a simpler modified production process that requires the repeated alteration of the centrifugation speed [5]. Consequently, its relatively dense structure is more comparable to that of natural fibrin and involves sample amounts of growth factors and proteins that are derived from autologous platelets and leukocytes. The dentin-pulp complex (DPC), consisting of dentin and dental pulp, originates from the dental papilla of the tooth germ during embryogenesis and has interrelated functions [41]. Odontoblasts in the dental pulp form dentin bridges in response to physiological or pathological stimuli, including pathogens, thus serving a defensive function [42]. While essential for the long-term integrity and vitality of teeth, the dentin-pulp complex is sensitive to damage caused by external factors. Due to the various limitations of traditional approaches for preserving the dentin-pulp complex, there is a need for new methods for its restructuring [18, 43]. CGF, a platelet concentrate, holds promise as a scaffold for the treatment of dentin-pulp complex disorders. As a biomaterial with a rich combination of growth factors and chemotactic factors, CGF may serve as a promising biomaterial in clinical applications for promoting pulp regeneration due to its excellent regulatory properties in inflammation, proliferation, migration, and odonto/osteogenic differentiation [18, 20]. No studies are addressing the effect of pulp damage induced by CGF pulpotomy. In addition, studies are reporting that CGF increases periodontal regeneration in clinical studies [44]. Öngöz Dede et al. [45]. demonstrated that CGF contributed to gingival regeneration by increasing the gingival thickness. In addition, Firouzi et al. [46] reported that CGF enhanced bone regeneration induced by lipopolysaccharide in cell culture. Similarly, in this study, we observed that CGF regenerated pulpotomy-induced damage. In current studies, although the effects of CGF on tissue regeneration are available, the information about its effect on tissue damage mechanisms is not clear. In their study, Xu et al. [18] investigated the effects of CGF on proliferation, migration, and differentiation in human dental pulp stem cells (hDPSCs) exposed to lipopolysaccharide (LPS) in vitro, as well as the potential role of CGF in pulp regeneration in immature teeth in vivo. The results of this study revealed that CGF not only inhibited the release of proinflammatory cytokines such as TNF-α but also promoted in vitro proliferation, migration, and odonto/osteogenic differentiation of LPS-stimulated hDPSCs. They concluded that CGF supports the regeneration of the dentin-pulp complex, suggesting it is a promising alternative biomaterial in regenerative endodontics. However, in this study, the TNF-α level in the CGFG group is higher compared to the control group, and it did not show a significant decrease from the second week to the fourth week. This difference can be explained by the variability in experimental conditions and events triggering inflammation. In Xu’s study [18], the inflammatory process was triggered by LPS, while in the current study, it was triggered by pulpotomy. In the study by Yuan et al. [47]. , they aimed to investigate the effect of let-7c-5p (down-regulated in dental pulp tissues in inflammatory disorders) on LPS-induced pulpitis. They attempted to explain this by focusing on the expression of dentin matrix protein-1 (DMP1) and the NF-kβ pathway induced by LPS. They indicated that LPS application increased the production of IL-1β and TNF-α and reduced the viability of dental pulp stem cells by increasing DMP1 expression and activating the NF-kβ pathway. In this current study, similar to the study by Yuan et al. [47]. , the proinflammatory cytokine mechanism increased in induced inflammatory reactions due to pulpotomy, showing an increase in TNF-α and NF-kβ/p65 levels compared to the control group. Kamal et al. [48]. reported that CGF application reduced inflammation and pain induced by low-dose laser application. In this current study, however, in the CGFG group, there were more edematous and inflammatory areas compared to the control group. Moreover, TNF-α and NF-kβ/p65 levels were increased compared to CG. In the study by Kamal et al., the role of CGF in wound healing was evaluated in the inflammatory process caused by alveolar osteitis and was found to be successful. However, in the current study, the differences in the condition causing inflammation and the therapeutic agent used may explain the differences in the results.

MTA has been adopted as an endodontic material, especially as a pulp capping material, for longer than a decade; however, certain biological effects on pulp tissue regeneration remain unknown [10]. Silva et al. [22]. , reported that the cytokines IL-1, IFN-, and the chemokine CCL5 in the pulp tissue of rat incisors were modified when exposed to MTA. Inflammatory cytokines and chemokines play a role in the aggregation of leukocytes towards injured or infected tissues; however, they may exacerbate tissue damage as well. Tohma et al. [34]. employed the MTA pulpotomy model for 14 days. The results of the study revealed a scar-healing pattern that was generally consistent with previous studies. Similarly, Prabhakar et al. [49] reported that the inflammation and edema caused by pulpotomy-related pulpitis in rats decreased after MTA application. They reported an improvement in tissue structure on days 14 and 30 after the pulpotomy. Salako et al. [32]. reported that MTA reduced the inflammation resulting from pulpotomy-induced pulpitis in 14 days. Moreover, they determined that inflammation was further reduced by MTA on day 30 compared to day 14. Similarly, we observed that the MTA treatment reduced the inflammation encountered in pulpotomy-induced pulpitis in 14 days, and that this reduction was more pronounced on day 30. A study by Benetti et al. [50]. on dental root canal treatment reported MTA to exert an anti-inflammatory effect by causing a decrease in TNF-α expression. Similarly, in the present study, we found that the MTA treatment reduced the pulpotomy-induced increase in TNF-α positivity. Moreover, we demonstrated a further decline in cells showing TNF-α positivity on day 30 compared to day 14. However, although the effects of MTA on the nuclear factor-kappa B signaling system have not been previously researched, MTA can cause the initial inflammation of rat dental pulp cells through its effects on the NF-kβ/p65 signaling system, while a subsequent anti-inflammatory effect is also observed due to the regulation of the expression of certain inflammatory mediators. Minic et al. [33]. revealed that it stimulates the toll-like receptor 4 found on dental pulp cells and produces inflammatory cytokines such as IL-1β and IL-6 by activating the NF-kβ pathway in pulpotomy-induced pulpitis.

In this study, we observed an increase in cytokines, specifically TNF-α and NF-κβ/p65 pathways, during the inflammatory process induced by pulpotomy compared to the control group. Both MTA and CGF pulpotomy treatments triggered cytokines in the inflammatory process. When comparing the study groups, MTA material induced the proinflammatory pathway less than CGF, and the positivity of TNF-α and NF-κβ/p65 in this pathway decreased over time. Different doses and durations of proinflammatory cytokines may have varying effects on dental pulp cells. Chronic exposure of dental pulp cells to TNF-α (> 3 days) impairs the differentiation ability of odontoblasts, which suggests that inflammatory cytokines may hinder the repair and regeneration of dentin and pulp during inflammation [51, 52]. In a study by Mimic et al. [33], using Biodentin, a material similar in structure to MTA, they observed an increase in TNF-α levels after pulpotomy, similar to these findings. Xu et al. [18], they determined that CGF not only inhibits the release of proinflammatory cytokines such as TNF-α but also promotes in vitro proliferation, migration, and odonto/osteogenic differentiation of LPS-stimulated hDPSCs. Pribadi et al. [29], where they analyzed the effect of calcium hydroxide and propolis combination on pulpal injury, they stated that this combination inhibits pulpal inflammation by reducing NF-κβ expression and collagen type 1 on dental pulp. Therefore, this combination has been suggested as a promising new material for pulpal treatment. The different effects of materials used in numerous studies on proinflammatory pathways can be explained by differences in experimental conditions and induced pulpal inflammation pathways.

First, this study is a pilot study considering the effects of second-generation autologous growth factors on pulpotomy-induced inflammation by tissue levels of TNF-α and NF-kβ/p65. In general, along with limitations specific to animal models, This study also had some limitations particular to This research. This study focused on comparing pulpotomy treatments using MTA and CGF. There is a need for additional studies supported by positive control groups where only pulpotomy is performed without the use of therapeutic agents. The present study should be supported by studies dealing with other inflammatory cytokines and cell signalling molecules. Similarly, this study needs to be corroborated by research on specific cytokines, and intracellular and mitochondrial calcium levels that address cell damage mechanisms.

## Conclusion

In conclusion, this study revealed a decrease in TNF-α and NF-kβ/p65 positive cells in the MTA treatment group compared to the CGF group. Furthermore, observed a reduction in the number of cells exhibiting TNF-α and NF-kβ/p65 positivity in the 4th week following MTA application compared to the 2nd week. While MTA demonstrated more favourable outcomes compared to CGF within the scope of This study, further experimental and clinical investigations are warranted to garner more conclusive data regarding CGF.

## Data Availability

The datasets used and/or analysed during the current study available from the corresponding author on reasonable request.
